# The World's Babies

**Published:** 1889-01-05

**Authors:** 


### BABIES.

### I.—THE WORLD'S BABIES.

It is calculated that somewhere on the face of the earth there is a baby born with every tick of the clock, rather more in fact; about seventy a minute some statisticians affirm, or a total of 36,792,000 every year.

How can we most clearly realise Nature's bounty in this one little matter of babies? Parcel out some great plain into a number of small plots, like a huge chessboard, with as many squares in it as there are men, women, and children in the whole of London. Into every square put about seven babies, and you have something like the world's annual produce displayed. Put the little cherubs each in a crib a yard wide, and stand the cribs side by side close together, and you will have a line of them that will reach very nearly round the world, a line considerably over 20,000 miles long at all events.

Again, supposing them all to grow up, and the sexes to be about equally divided, they would make an army a hundred times as large as the entire military force of Great Britain at home and abroad, and every soldier might have a wife.

Let us imagine that some Pied Piper of Hamelin has piped his way through the world, and has drawn all last year's babies together in one vast assembly, and has arranged them in a solid circular mass, allowing a square yard for each child. Take your stand in the midst—don't be nervous; probably not more than twenty-five per cent. of them will be lifting up their voices to weep at any one time—take your stand in the midst of this interesting little ecumenical assembly, and you may look out in any direction over nearly two miles of babies!

And what a gathering it will be! How many shades of colour; how many varieties of form; how many types of beauty, aye, and of ugliness; how many diversities of nature! What differences of climate and conditions of life and habits they represent! How many various phases of civilisation and savagery! Young as they are, what perils they have escaped from wild beasts and savage warfare, and equally savage religious and social customs, and medicine-men, and barbarous mothers, and slave-hunters.

A very miscellaneous gathering indeed is this. Here are royal babies, who, like the young king of Spain the other day, have been first introduced to society on massive gold trays, wrapped in swaddling-clothes, redolent of papal benedictions, and worth a small fortune; and here are babies that have made their *début* in London cellars, noisy with drunken curses, and have been dependent on the nearest maternal society for their first outfit. There are those who have given their earliest cry under the shade of some New Zealand bush, have been washed in some neighbouring stream, and forthwith slung upon the mother's back; and others of whose birth "the great cannon to the clouds" have told, while bells have rung and bonfires burned, and joyous pæans have gone up in gorgeous cathedrals. Many have been born only to find that for a good many hours in the day they must rub along as best they can in charge of a nurse only a little bigger than themselves while mother is out washing; and some have lighted on luxury such as that of the baby Grand Duke Michael Alexandrovitch, the youngest son of the Czarevitch, who, before he was many weeks old, is reported to have had the sum of £3,369 devoted to his first year's maintenance, and some fifteen people entirely set apart for his personal service. There are some as beautiful as the infant Haroun-al-Raschid, "whose face," his biographers tell us, "was like the full moon, whose eyes were like the stars Aisch and Kesil, and whose lips were like twin pomegranates;" others, such for instance, as yonder little Bosjesmans, who look more like magnified yellow toads than human babies. Stupendously thick in the body, with heads that look as though badly afflicted with water on the brain, and eyes that have squeezed up to exclude the sandfly, till they seem to have retired into the head, and are all but concealed by the projecting cheekbones and the puffy fat with which they are covered, the Bosjesman baby—to European eyes, at least—is a positively repulsive little creature.

Set them all out, nation by nation, tribe by tribe, and let us look at them. We would put them in their mothers' arms, or sling them on their backs, and make them file by; only that there is a practical difficulty in doing this, even in imagination. For if they filed along at the rate of twenty a minute, and we stood at our post of inspection twelve hours a-day for seven days in the week, it would take not less than about six years and a-half for us to see the whole of them. Not only would such a march past be somewhat tedious, but long before we had come near the end of it, many of the babies would have developed into ferocious little huntsmen, agile as monkeys, uncontrollable as zebras, and spiteful as ferrets.

Set them out therefore in one great gathering, and let us look over them, and notice a few of their characteristics. See yonder that vast mass of young Celestials with their parchment-looking skins, and almond-shaped eyes, and their little bald pates—every one of them scrupulously shaved from the time that it is a month old. Near them may, perhaps, be a smaller contingent not altogether unlike them—the babies of Japan—with heads also shaved, only that they have four queer little tufts of hair left on their crowns. Most of them, travellers tell us, have been habitually drenched in cold water and plunged in snow to make them hardy—just as historians affirm that Gustavus IV. of Sweden was ducked until the very life of the hapless little princeling was well-nigh ducked out of him. Round yonder, on the other side of the great assembly, are thousands of little mortals who have never yet been washed at all, and probably never will be. They are Esquimaux, born in a snow igloo, and have already, some of them, begun to fatten on whale-blubber, that will presently appear to ooze through their dusky little hides, which as yet have never been wrapped in anything but the furry hoods of their mother's cloaks and a continually-thickening layer of lamp black and grime acquired from day to day in their icy snuggeries. Esquimaux never wash at any period of their lives—don't at all understand the business, and think it amazingly funny to see anybody else go through so queer a performance. Even worse off in point of cleanliness are those hapless little Egyptians yonder. The mothers, poor souls, would like to wash them and smarten them up, but they have—at any rate, in the remoter parts of the country—a superstitious belief in the powers of the "evil eye," which will be the more likely to blight their little ones the prettier and more attractive they are. For a twelvemonth or so they will leave them, not only undressed and unwashed, but they will blacken their foreheads with soot, or bedaub them with clay, or keep them wrapped up in a black veil, and they will take it very unkind of any friend who shall speak in praise of their babies' beauty. To be considerate and polite a visitor who looks at the baby must say, "Dear me! What a fright!" or "What an ugly little thing!" Many a one over there among those Egyptian infants will be found to be a pitiable little object, filthy and neglected, and not infrequently blinded by soreness of the eyes, due to the irritation of the flies to which the little creatures have been exposed.

*(To be continued.)*

